# Sting Is Commonly and Differentially Expressed in T- and Nk-Cell but Not B-Cell Non-Hodgkin Lymphomas

**DOI:** 10.3390/cancers14051186

**Published:** 2022-02-24

**Authors:** Ioanna Xagoraris, Pedro Farrajota Neves da Silva, Georgia Kokaraki, Konstantina Stathopoulou, Björn Wahlin, Anders Österborg, Nikolas Herold, Siok-Bian Ng, L. Jeffrey Medeiros, Elias Drakos, Birgitta Sander, George Z. Rassidakis

**Affiliations:** 1 Department of Oncology and Pathology, Karolinska Institute, 17177 Stockholm, Sweden; ioanna.xagoraris@ki.se (I.X.); pedro.farrajota.neves.da.silva@ki.se (P.F.N.d.S.); georgia.kokaraki@ki.se (G.K.); konstantina.stathopoulou@ki.se (K.S.); anders.osterborg@ki.se (A.Ö.); 2 Department of Medicine, Karolinska Institute, 17177 Stockholm, Sweden; bjorn.wahlin@ki.se; 3 Department of Women’s and Children’s Health, Karolinska Institute, 17177 Stockholm, Sweden; nikolas.herold@ki.se; 4 Department of Pathology, Yong Loo Lin School of Medicine, National University of Singapore, Singapore 119074, Singapore; patnsb@nus.edu.sg; 5 Department of Hematopathology, The University of Texas MD Anderson Cancer Center, Houston, TX 77030, USA; ljmedeiros@mdanderson.org; 6 Department of Pathology, Medical School, University of Crete, 71110 Heraklion, Crete, Greece; hdrakos@hotmail.com; 7 Department of Laboratory Medicine, Karolinska Institute, 17177 Stockholm, Sweden; birgitta.sander@ki.se

**Keywords:** STING, T-cell non-Hodgkin lymphoma, immune response

## Abstract

**Simple Summary:**

T/NK-cell non-Hodgkin lymphomas (NHLs) represent approximately 10% of all NHLs and most patients have a poor outcome using current treatment options. Molecules involved in the host response against lymphoma cells are currently being investigated in an effort to develop novel therapeutic strategies combining targeted therapy and immunotherapy. In this study, we show that expression of STING, a key protein in the cGAS–STING immune response pathway, is restricted to lymphomas of T- and NK-cell origin and seems to be down regulated in B-cell NHLs. These results are based on the analysis of 14 lymphoma cell lines of various types at the RNA and protein level and immunohistochemical analysis of a large number of B-cell (*n* = 265) and T/NK-cell (*n* = 158) NHLs obtained from previously untreated patients from three institutions. In these patient cohorts, STING is differentially expressed among T/NK-cell NHLs, whereas all B-cell NHLs were negative for STING expression. Thus, STING represents a novel biomarker and therapeutic target in T- and NK-cell lymphomas with direct immunotherapeutic implications, since modulators of cGAS–STING activity are already available for clinical use, and could therefore be used to benefit patients with these difficult-to-treat diseases.

**Abstract:**

The expression patterns of stimulator of interferon genes (STING) were investigated in a cohort of 158 T- and natural killer (NK)-cell and 265 B-cell non-Hodgkin lymphomas (NHLs), as well as in control reactive lymph nodes and tonsils. STING expression was assessed by immunohistochemical methods using diagnostic biopsy specimens obtained prior to treatment. Using an arbitrary 10% cutoff, STING was differentially expressed among T/NK-cell NHLs; positive in 36 out of 38 (95%) cases of ALK+ anaplastic large cell lymphoma (ALCL), 23 out of 37 (62%) ALK-ALCLs, 1 out of 13 (7.7%) angioimmunoblastic T-cell lymphomas, 15 out of 19 (79%) peripheral T-cell lymphomas, not otherwise specified, 20 out of 36 (56%) extranodal NK/T-cell lymphomas of nasal type, 6 out of 7 (86%) T-cell lymphoblastic lymphomas, and 3 out of 4 (75%) mycosis fungoides. STING expression did not correlate with clinicopathological parameters or outcome in these patients with T/NK-cell lymphoma. By contrast, all 265 B-cell NHLs of various types were STING-negative. In addition, STING mRNA levels were very high in 6 out of 7 T-cell NHL cell lines, namely, ALK+ and ALK-ALCL cell lines, and very low or undetectable in 7 B-cell NHL cell lines, suggesting transcriptional downregulation of STING in neoplastic B-cells. At the protein level, using Western blot analysis and immunohistochemistry performed on cell blocks, STING expression was found to be restricted to T-cell NHL cell lines. Taken together, STING expression represents a novel biomarker and therapeutic target in T- and NK-cell lymphomas with direct immunotherapeutic implications since modulators of cGAS–STING activity are already available for clinical use.

## 1. Introduction

In their normal state, cells need to distinguish between their own DNA localised in the nucleus and genetic material from pathogens, such as microbes or viruses, that are encountered in the cytosol. As a protective mechanism, cells normally respond to cytoplasmic DNA by activating an inflammatory response. However, in neoplastic cells host DNA can also accumulate in the cytoplasm. Cytosolic DNA of either endogenous or exogenous origin is sensed by the cGAS–STING pathway to activate innate immune responses. Cyclic GMP–AMP (cGAMP) synthase (cGAS) is a cytosolic DNA sensor that activates innate immune responses through production of a second messenger, cGAMP, which then activates the adaptor protein STING. The latter activates the transcription factors IRF3 and NF-κB through phosphorylation by the kinases TBK1 and IKK, respectively. Activated IRF3 and NF-κB translocate into the nucleus to elicit the expression of interferons (IFNs) and other cytokines [1].

T-cell and NK-cell non-Hodgkin lymphomas (NHLs) represent approximately 10% of all NHLs and affected patients mostly have a poor outcome using current treatment modalities. In the World Health Organization (WHO) classification, systemic T/NK-cell neoplasms are grouped into nodal and extranodal lymphomas. Nodal lymphomas include anaplastic lymphoma kinase (ALK)-positive anaplastic large cell lymphoma (ALCL); ALK-negative ALCL; angioimmunoblastic T-cell lymphoma (AITL); and peripheral T-cell lymphoma, not otherwise specified (PTCL-NOS). Extranodal lymphomas include natural killer (NK)/T-cell lymphoma of nasal type (NK/T-nasal type); intestinal T-cell lymphoma types; as well as primary cutaneous lymphomas, such as mycosis fungoides (MF) [2]. The incidence of T/NK-cell lymphomas varies geographically [3]. Although certain types of T/NK-cell lymphoma are characterized by a defining genetic abnormality (i.e., ALK chromosomal translocations in ALK+ ALCL), disease-specific genetic alterations are generally lacking in other histological types of T/NK-cell lymphoma, despite certain reported associations [4,5,6,7]. Therefore, it is crucial to identify disease-specific biomarkers among T/NK-cell lymphomas, thereby facilitating the design of personalized therapeutic approaches for affected patients.

The survival of patients with T/NK-cell lymphomas is generally poor because of treatment failure, with the exception being younger patients with ALK+ ALCL. Patients with T/NK-cell lymphomas may be refractory to primary therapy or may respond but subsequently relapse, with both groups associated with a dismal outcome, despite intensification of chemotherapy [3,8]. Since most types of T/NK-cell lymphomas are associated with a dismal prognosis [9], there is an urgent need for development of new targeted therapies and possible immunotherapy approaches.

The expression patterns of stimulator of interferon genes (STING) in NHLs are unknown to date. In the present study, we investigated for the first time STING expression in well characterized cohorts of patients with common types of T/NK-cell and B-cell NHL. We report that STING expression is restricted to T/NK-cell lymphomas, and thus represents a novel marker and target of therapy in patients with these lymphomas.

## 2. Results

### 2.1. Expression of STING mRNA and Protein in NHL Cell Lines

**Figure 1:**
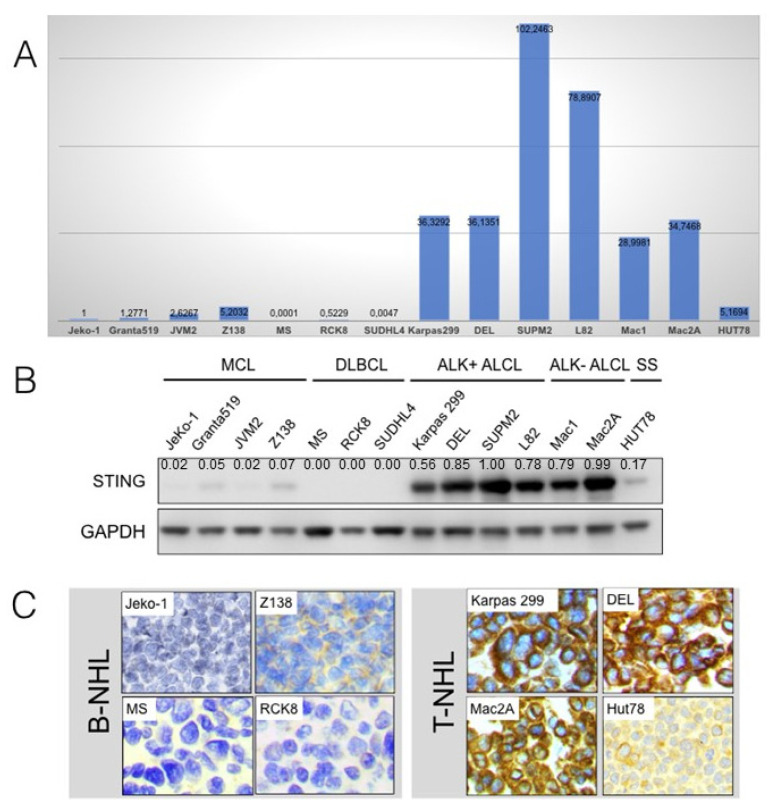
STING gene and protein expression in B- and T-cell non-Hodgkin lymphoma cell lines. (**A**) Real-time quantitative PCR performed on 14 cell lines of various B- and T-cell non-Hodgkin lymphoma (NHL) types showed STING gene expression in T-cell but not in B-cell lymphomas. Among T-cell NHLs, ALK+ and ALK-ALCL cell lines demonstrated the highest STING RNA levels, whereas the HUT-78 cell line (Sezary syndrome) showed low levels. (**B**) Using Western blot analysis performed on the same cell lines, strong expression of STING protein was found only in T-cell but not in B-cell lymphomas. (**C**) The Western blot findings were confirmed by immunohistochemistry using the same antibody performed on cell blocks prepared from the same cell lines. Expression of STING was cytoplasmic in all positive cells. Original magnification ×400; DAB as chromogen.

STING mRNA and protein levels were assessed in 14 B- and T-cell NHL cell lines (Figure 1). Real-time quantitative PCR (RT-PCR) showed expression of STING transcripts in T-cell lymphoma cell lines, whereas STING mRNA level was undetectable or extremely low in B-cell lymphoma cell lines. Among T-cell NHLs, ALK+ and ALK-ALCL cell lines had the highest STING mRNA levels, whereas the HUT-78 cell line (Sezary syndrome) showed low levels. The RT-PCR findings were strongly associated with the Western blot data performed on the same 14 cell lines that demonstrated strong expression of STING at the protein level only in T-cell lines. The Western blot analysis findings were further confirmed by immunohistochemistry performed on formalin-fixed, paraffin-embedded cell blocks prepared from the same cell lines using the same STING antibody. Expression of STING was cytoplasmic in all STING-positive cell lines.

### 2.2. STING Expression in Reactive Lymph Nodes and Tonsils

**Figure 2:**
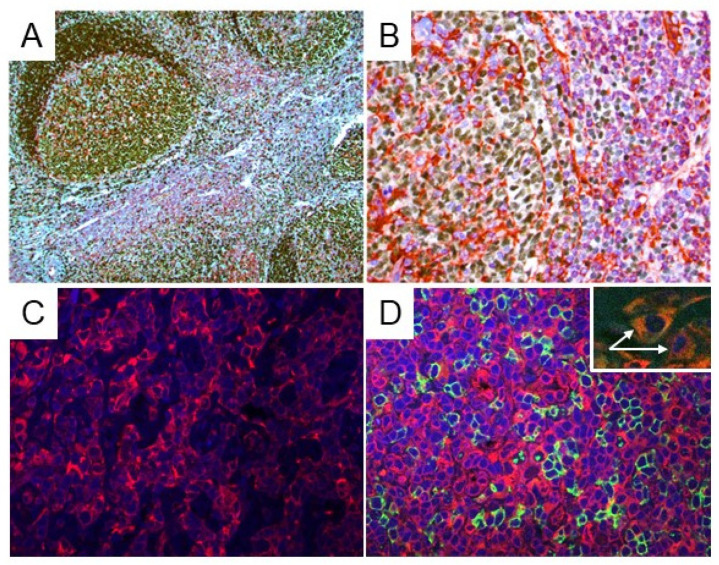
STING expression in reactive lymph nodes and tonsils. (**A**,**B**) Double immunostaining for STING (red) and PAX5 (dark brown) was performed in reactive lymph nodes and tonsils (free of malignancy). STING expression was observed in dendritic cells, histiocytes, and in a subset of reactive, small T-lymphocytes. The B-lymphocytes of the germinal centers were all STING-negative. Original magnification: (**A**) ×100; (**B**) ×400. (**C**,**D**) Indirect immunofluorescence for STING (red) and CD20 (green) was performed on reactive lymph nodes and tonsils: (left panel) STING expression is present in the T-cell area of the reactive lymph node (interfollicular area); (right panel) CD20+ B lymphocytes (green) are all negative for STING expression (red), which is restricted to T-lymphocytes. Two histiocytes (white arrows) positive for CD68 (green) and STING (red) are shown in the inset, as well.

In all reactive lymph nodes and tonsils (free of malignancy), STING was expressed in dendritic cells (Figure S1), histiocytes, and a subset of small reactive T-lymphocytes that served as internal positive controls for STING staining in all cases. STING was detected in the cytoplasm of reactive T-lymphocytes with variable staining intensity (Figure 2). No STING protein expression was observed in reactive B-cell populations.

### 2.3. STING Expression in T-Cell and B-Cell NHLs

**Figure 3:**
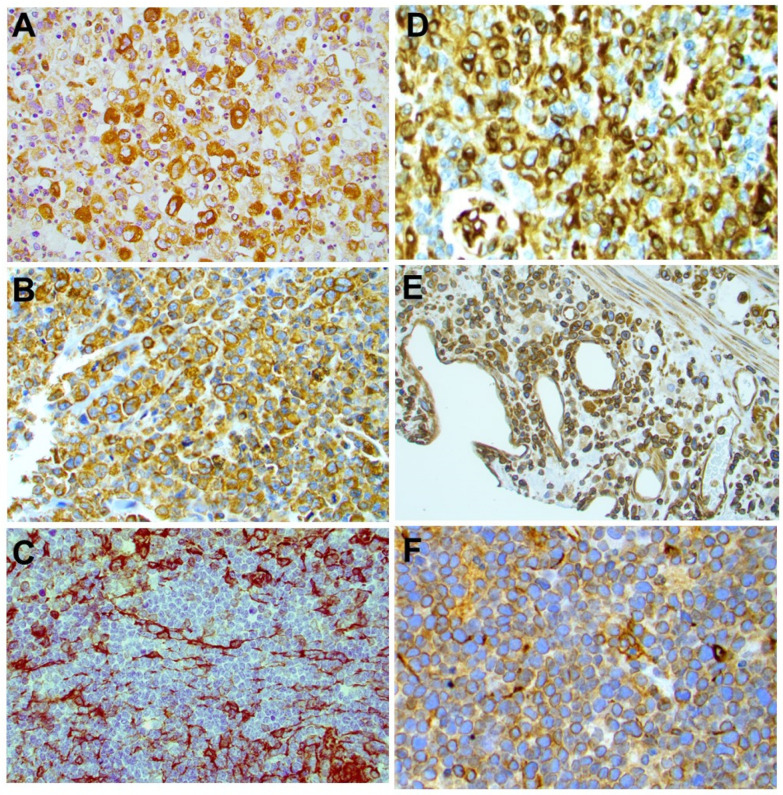
Expression of STING in different types of T-cell lymphomas. STING was differentially expressed among PTCL histological types. Representative examples of STING-positive ALK+ ALCL (**A**), ALK-ALCL (**B**), PTCL-NOS (**D**), NK/T-nasal type (**E**), and T-LBL (**F**) are shown. A representative STING-negative AILT case (**C**) is also shown. Original magnification ×400 (**A**–**D**,**F**) and ×200 (**E**). DAB as chromogen.

**Figure 4:**
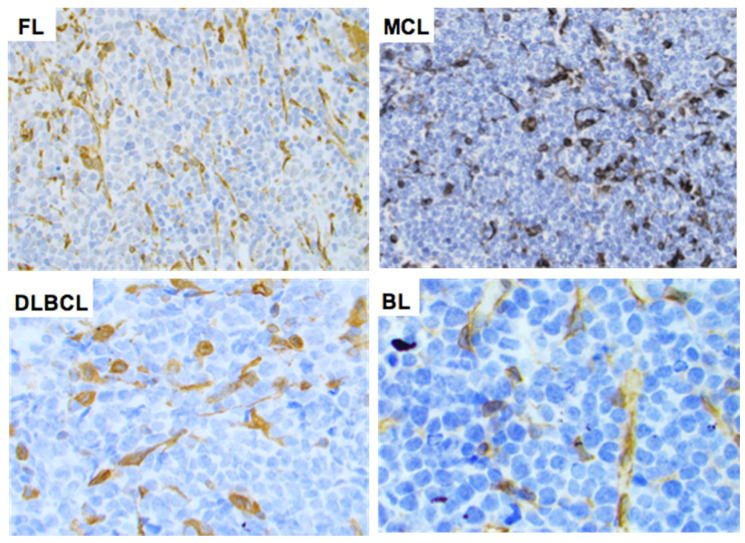
Expression of STING in B-cell non-Hodgkin lymphomas. STING expression was negative in the neoplastic cells of all B-cell NHL tumors assessed. Representative examples of follicular lymphoma (FL), mantle cell lymphoma (MCL), diffuse large B-cell lymphoma (DLBCL), and Burkitt lymphoma (BL) are shown. Original magnification ×400. DAB as chromogen.

**Figure 5:**
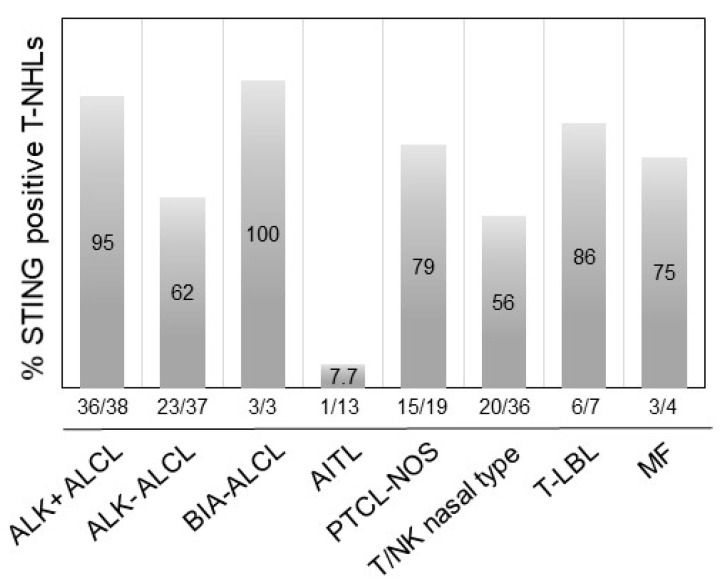
Distribution of STING expression frequency among common histological types of T/NK-cell lymphomas. STING expression was most frequent in BIA-ALCL, ALK+ ALCL, and T-LBL, followed by other T/NK-cell NHLs. The difference in the frequency of STING expression among the histological types of T/NK-cell lymphoma was statistically significant (*p* < 0.001 by chi-square test).

Using an arbitrary 10% cutoff, STING was positive in 36 out of 38 (95%) cases of ALK+ ALCL, 23 out of 37 (62%) ALK-ALCL, 3 out of 3 (100%) breast implant-associated (BIA) ALCL, 1 out of 13 (7.7%) AITL, 15 out of 19 (79%) PTCL-NOS, 0 out of 1 (0%) MEITL, 20 out of 36 (56%) NK/T-nasal type, 6 out of 7 (86%) T-LBL, and 3 out of 4 (75%) MF cases (Figure 3). The difference in the frequency of STING expression among the different types of lymphoma is significant (*p* < 0.001 by chi-square test). No significant associations between STING expression and patient characteristics in the entire patient group or separately in the T/NK-cell lymphoma types was observed (not shown). By contrast, all 265 cases of B-cell NHL were negative for STING expression (Table 1, Figure 4 and Figure 5). In these neoplasms, dendritic cells and a variable number of reactive T-lymphocytes were positive, thus serving as internal positive controls for all tissue specimens. To further confirm the absence of STING expression in B-cell NHLs, double immunostaining for STING and the pan-B-cell marker PAX5 was performed on a large subset of follicular lymphomas (FL) included in a tissue microarray (TMA). All FL tumor cells were STING negative (Figure S2).

### 2.4. Survival Analysis

Univariate and multivariate survival analyses were performed for the entire group of T/NK-cell lymphomas and separately for certain types with sizeable STING expression, including ALK-ALCL, AITL, PTCL-NOS, and NK/T-nasal type. Superior freedom from progression (FFP) and overall survival (OS) were observed in patients with ALK+ ALCL compared with other histological types, thus confirming previously reported data (Figure S3). STING expression was not correlated with FFP or OS in any of the T/NK-cell histological types. Multivariate survival analysis in the study cohort was assessed using a Cox proportional hazards model and showed no significant associations between patients with STING-positive and STING-negative tumors in any type of T- or NK-cell lymphoma.

## 3. Discussion

We report here that STING expression is restricted to T-cell lymphoma cell lines and T/NK-cell lymphomas among NHLs. Using a panel of T- and B-cell NHL cell lines and a quantitative RT-PCR assay, we show that STING mRNA levels were undetectable or extremely low in B-cell neoplasms. By contrast, most T-cell lymphoma cell lines showed very high gene expression at the RNA level, with the highest levels being observed in the ALK+ and ALK-ALCL cell lines. These results were confirmed at the protein level by both Western blot analysis and immunohistochemistry performed on corresponding formalin-fixed, paraffin-embedded cell blocks. Similarly, T/NK-cell lymphomas of various histological types commonly and differentially express STING in the neoplastic cells. In contrast, all B-cell lymphomas assessed in this study were negative for STING.

Our data suggest that STING may represent a novel marker for NHLs of T- or NK-cell lineage which can be used as a diagnostic marker for certain T-cell lymphomas with “null” cell immunophenotype (i.e., “null” cases of ALK-ALCL). The difference in STING expression between AITL and PTCL-NOS is of particular interest. Previous studies have reported genetic alterations, such as RhoA-GTPase mutations that are more frequent in AITL compared with PTCL-NOS [5,6]. However, PTCL-NOS with angioimmunoblastic-like features may carry these mutations as well. Our findings, showing that only 7.7% of AITLs are positive for STING as compared to 79% of PTCL-NOS, provide a novel immunohistochemical marker that could be helpful in distinguishing between these two lymphoma types that may share similar morphological features, although the existing immunohistochemical and molecular methods are quite efficient for classification. Since the number of patients in the AITL and PTCL-NOS groups is rather limited, larger cohorts are needed to confirm the substantial difference in STING expression between these two neoplasms. To the best of our knowledge, this is the first study to show the expression patterns of STING in T- and NK-cell lymphomas.

The mechanism(s) underlying silencing of STING gene expression in B-cell NHLs merits further investigation. In a previous report by Gram et al., STING protein was absent in primary B-cells (peripheral blood mononuclear B-cells) and EBV-negative B-cell lines, yet present in EBV-transformed B-lymphoblastoid cell lines [10]. However, B-cell lines expressed other key components of the cytoplasmic DNA-sensing cGAS–STING pathway, which seems to be activated even with low expression levels or in the absence of STING, possibly by alternative mechanisms [10]. In the same study, normal human B-cells failed to secrete type I interferons upon cytoplasmic DNA exposure and this finding was attributed to a dysfunctional cGAS–STING pathway [10]. These findings further support our data on B-cell lymphomas and, taken together, suggest that a still unknown mechanism may silence or at least downregulate STING expression in normal and neoplastic EBV-negative B-cells. Moreover, our findings showing no STING expression in all B-cell NHLs included in this study may indicate the limited biological significance of tumor cell-producing STING in these tumors or alternative mechanisms of anti-tumor responses. However, in a recent study, Tang et al. [11], using STING-deficient and STING-proficient non-malignant B-cells and a chronic lymphocytic leukemia mouse model, showed that STING-deficient cells were indeed more responsive to B-cell receptor activation than their STING-proficient counterparts. Their results point to the negative regulation of B-cell receptor signaling in both normal and malignant murine B-cells [11]. However, these investigators did not assess STING expression in human B-cells in their study. In an earlier study, Walker et al. [12] showed that B-cells can be activated directly by cyclic dinucleotides in a STING-dependent manner in vitro and in vivo and that BCR and STING signaling pathways act synergistically to promote antibody responses. However, the relative levels of STING expression between normal or neoplastic B- versus T-cells were not assessed [12].

Regulation of STING expression and function may be complex. For instance, loss-of-function STING gene mutations have been reported in several tumor types, which may result in suppression of STING signaling [13]. Examination of reported missense STING variants confirmed that many exhibit a loss-of-function phenotype and cannot activate cytokine production following exposure to cytosolic DNA or DNA-damage events. In addition, epigenetic mechanisms involving the cGAS and STING genes have been reported to reduce their function [13,14]. In a recent study on melanoma, using genome-wide DNA methylation profiling, investigators showed that promoter hypermethylation of the cGAS and STING genes mediates their coordinated transcriptional silencing and contributes to the widespread impairment of the STING signaling function [14]. In addition, important transcription factors, such as CREB and MYC, may be involved in transcriptional regulation of the STING gene [15]. Furthermore, Ma et al. [16] identified a STAT1 binding site in the STING promoter that contributes to activation of STING transcription.

The cGAS–STING signaling pathway transfers signals from the binding of ligands and receptors to activate TBK1 and IKK kinase, leading to activation of important transcription factors, such as IRF-3 and NF-kB. The latter induce production of type I interferons (IFNs) to elicit anti-tumor immunity [17]. Whether tumors can afford cGAS–STING pathway activation by generating a T-cell-rich tumor microenvironment has therapeutic implications. In many mouse models of cancer (e.g., breast cancer, colon cancer, melanoma, etc.), injecting cGAMP results in the accumulation of macrophages that secrete tumor necrosis factor (TNF)-alpha and chemokines, thereby recruiting T-cells and suggesting a role for macrophages in STING-induced anti-tumor effects [18]. In addition, recent in vivo studies employing B-cell lymphoma models and wild-type or STING knockout hosts bearing either wild-type or STING knockout tumor cells, respectively, showed that tumor regression was dependent exclusively on STING expression by the tumor microenvironment [19]. Thus, STING adjuvant administration may augment immune-mediated antitumor response, independent of STING pathway activation status in cancer cells [19].

Since STING agonists promote immune responses, co-injection of immune checkpoint inhibitors (anti-CTLA-4 and anti-PD-1) may further enhance anti-tumor effects [20,21,22]. In a recent preclinical study, a STING agonist combined with agents that improve APC or T-cell function enhanced the control of distant lymphoma tumors by modulating anti-tumor immune responses. These data provide a rationale for the therapeutic use of STING agonists followed by anti-PD-1 antibodies as immunotherapy for human lymphomas [22]. Indeed, current clinical trials are based on a combination of cGAS–STING pathway agonists with immune checkpoint inhibitors, such as pembrolizumab, ipilimumab, and nivolumab, for cancer immunotherapy. Enhancing the immune system’s ability to fight against cancer by delivering potent STING agonists with appropriate delivery systems can increase the efficacy of combinational cancer therapeutic strategies [17]. In addition, future studies should investigate the prognostic and predictive significance of STING pathway proteins and their regulators in large cohorts of patients with various T-cell lymphoma types.

## 4. Materials and Methods

### 4.1. Cell Lines

Fourteen NHL cell lines were used in this study, as listed in Supplementary Table S1, including seven T-cell and seven B-cell NHL cell lines. All cell lines were grown in Roswell Park Memorial Institute (RPMI)-1640 medium (Life Technologies, Grand Island, NY, USA) supplemented with 10% fetal bovine serum and incubated at 37 °C in a humidified atmosphere containing 5% CO_2_. Cell pellets were prepared using 20 × 10^6^ cells and the pellets were fixed in 4% formalin for 24 h and then embedded in paraffin to make cell blocks. Sections were cut from the cell blocks and the sections were subjected to immunohistochemistry analysis.

### 4.2. Gene Expression Analysis

Part of the collected cells were used for gene expression analysis. RNA was isolated and purified using a PureLink^TM^ RNA Mini Kit (Invitrogen/Thermo Fisher Scientific, Waltham, MA, USA), following the manufacturer’s instructions. The RNA concentration in individual RNA samples was determined using an Invitrogen^TM^ Qubit^TM^ RNA BR Assay Kit. Using a High-Capacity RNA-to-cDNA^TM^ Kit (Applied Biosystems/Thermo Fisher Scientific), 1 μg of RNA was reverse-transcribed. Generated cDNA was subjected to quantitative real-time PCR. Reactions were performed in triplicate using QuantStudio 7 Flex Real-Time PCR (Applied Biosystems/Thermo Fisher Scientific). To assess gene expression of STING we used the TaqMan primer/probe set for human TMEM173 (Thermo Fisher Scientific #Hs00736955_g1) and GAPDH (Thermo Fisher Scientific #Hs02786624_g1). STING was normalized to the housekeeping GAPDH gene and the relative expression was calculated, converting the difference in cycle thresholds (∆Ct) using the 2−∆Ct method.

### 4.3. Western Blot Analysis

Cells were collected during the exponential phase of growth, washed twice in cold PBS, and lysed at 4 °C in lysis buffer. Western blot analysis was performed using standard methods, as reported previously [23]. The primary antibodies used for this study were anti-STING (cat. no. 13647, Cell Signaling Technology, Leiden, The Netherlands), used at a dilution of 1:1000, and GAPDH (cat. no. 39-8600, Thermo Fisher Scientific, Stockholm, Sweden), used at a dilution 1:5000.

### 4.4. Patient Groups

The study cohort included 158 T- and NK-cell and 265 B-cell NHLs (Table 1). Among T-cell NHLs, the histological types assessed included ALK+ ALCL (*n* = 38), ALK-ALCL (*n* = 37), BIA-ALCL (*n* = 3), AITL (*n* = 13), PTCL-NOS (*n* = 19), monomorphic epitheliotropic intestinal T-cell lymphoma (MEITL) (*n* = 1), NK/T-nasal type (*n* = 36), T-lymphoblastic lymphoma (T-LBL) (*n* = 7), and MF (*n* = 4). Ethical approval for the use of patient tissues was obtained by the Institutional Research Board at each participating institution. Eligible patients had tissue specimens available for immunohistochemical determination of STING expression. The diagnosis and sub-classification of the NHL types were established according to criteria defined in the World Health Organization classification (2017) [2].

### 4.5. Therapy

Treatment included various regimens in accordance with protocols applied in the participating institutions, as specified in previous reports [9]. Clinical stage was determined according to Ann Arbor criteria. Anemia was defined as hemoglobin <13 g/dL and <11.5 g/dL for males and females, respectively. White blood cell count, lymphocyte count, erythrocyte sedimentation rate, and serum albumin level were not taken into consideration because of a high rate of missing values.

### 4.6. Immunohistochemical Methods

STING expression was assessed by immunohistochemistry using diagnostic biopsy specimens obtained prior to treatment. In addition, full tissue sections from five reactive lymph nodes and two tonsils were included as controls. All immunohistochemical analyses were performed in the same research laboratory (Karolinska Institute) using an identical protocol for all tumor samples, as previously described [24]. A monoclonal rabbit anti-STING antibody (cat. no. 13647, Cell Signaling Technology) and the UltraVision LP Detection System, Large Volume HRP Polymer (RTU) (Thermo Fisher Scientific, Stockholm, Sweden) were utilized. The specificity of the STING monoclonal antibody was tested previously by Western blot analysis and in the present study using cell blocks by immunohistochemistry. STING expression analysis was restricted to the neoplastic cells and positivity was defined as any level of unequivocal staining irrespective of intensity. In reactive lymph nodes and selected cases of FL, double immunostaining for STING and PAX5 (Roche) was performed using the Ventana robotic autostainer (Ventana, Tucson, AZ, USA). Evaluation of the immunostained slides was performed independently by two hematopathologists who were blinded to any clinical data. Based on the distribution of the proportions of STING-positive neoplastic cells (histogram), an arbitrary 10% cutoff was used to dichotomize STING expression.

### 4.7. Indirect Immunofluorescence

Co-expression of STING with CD3, CD20, and CD68 markers of T-lymphocytes, B-lymphocytes, and histiocytes (macrophages), respectively, was assessed by double indirect immunofluorescence and confocal microscopy in control lymphoid tissue specimens, including two lymph nodes and two tonsils, which were formalin-fixed and paraffin-embedded. The antibodies used were as follows: anti-STING antibody (rabbit, cat. no. 13647, Cell Signaling Technology, Danvers, MA, USA), CD3 (mouse, cat. no. 300406, BD Biosciences, Stockholm, Sweden), CD20 (mouse, cat. no. M075501-2, Dako, Agilent Technologies, Santa Clara, CA, USA), CD68 (mouse, cat. no. M087601-2, Dako, Agilent Technologies, Santa Clara, CA, USA). Briefly, after deparaffinization and rehydration of the tissue sections and antigen retrieval using a cooking steamer and antigen retrieval solution (cat. no. AR900250ML, Akoya Biosciences, Marlborough, MA, USA), the sections were incubated with the primary antibodies overnight at 4 °C followed by a 1h incubation with fluorescent secondary anti-rabbit (Alexa Fluor, cat. no. A-21207, Thermo Fisher Scientific, Stockholm, Sweden) and anti-mouse (Alexa Fluor, cat. no. A-11017, Thermo Fisher Scientific, Stockholm, Sweden) antibodies at room temperature. Mounting medium and DAPI (Vectashield, cat. no. H-1500, Vector Laboratories, Burlingame, CA, USA) were used as counterstain. Visualisation of immunofluorescence was performed using a confocal microscope (Zeiss LCM700, Jena, Germany).

### 4.8. Statistical Analysis

The associations of STING expression as a dichotomous variable with presenting clinical and laboratory features were assessed using the chi-square or Fisher’s exact tests as appropriate. Freedom from progression (FFP) was measured from the beginning of treatment to disease progression, relapse, or last follow-up [24]. Deaths from unrelated causes without prior disease progression or relapse were censored in the FFP analysis. Overall survival (OS) was measured from the beginning of treatment to the time of last follow-up or death from any other cause. Survival was visualized using Kaplan–Meier curves, and statistical comparisons between groups of patients were performed using the log-rank test. Cox proportional hazards models were used to evaluate the significance of STING expression after adjustment for other covariates. All statistical analyses were performed using the StatView statistical programme (Abacus, Berkeley, CA, USA).

## 5. Conclusions

We have shown that STING expression is restricted to T/NK-cell lymphomas. The lack of STING expression in normal and neoplastic B-cells may be due to downregulated transcription of the gene. Our results may have direct therapeutic implications because modulation of STING activity by STING agonists is being tested in ongoing clinical trials of patients with hematological malignancies [17]. In these trials, STING agonists are being combined with standard chemotherapy or immunotherapy with immune checkpoint inhibitors and the results may provide additional therapeutic strategies for patients with difficult-to-treat T/NK-cell malignancies.

## Figures and Tables

**Table 1:** Expression of STING in B-cell and T-cell non-Hodgkin lymphomas.

Lymphoma Type	Number of Patients	STING-Positive (*n*, %)
T-NHLs	158	107 (68%)
ALK+ALCL	38	36 (95%)
ALK-ALCL	37	23 (62%)
BIA-ALCL	3	3 (100%)
AITL	13	1 (7.7%)
PTCL-NOS	19	15 (79%)
MEITL	1	0 (0%)
NK/T-nasal type	36	20 (56%)
T-LBL	7	6 (86%)
MF	4 (2 typical, 2 transformed)	3 (75%)
B-NHLs	265	0 (0%)
FL	84	0 (0%)
MCL	72 (65 typical, 7 blastoid)	0 (0%)
MZL	19	0 (0%)
CLL/SLL	19	0 (0%)
DLBCL	61	0 (0%)
PMLBCL	4	0 (0%)
BL	3	0 (0%)
HG-NHL	3	0 (0%)
TOTAL	418	

Abbreviations: NHLs, non-Hodgkin lymphomas; ALK+ALCL, ALK+, anaplastic large cell lymphoma; ALK-ALCL, ALK–, anaplastic large cell lymphoma; BIA-ALCL, breast implant-associated-anaplastic large cell lymphoma; AITL, angioimmunoblastic T-cell lymphoma; PTCL-NOS, peripheral T-cell lymphoma, not otherwise specified; MEITL, monomorphic epitheliotropic intestinal T-cell lymphoma; NK/T-nasal type, extranodal T/NK-cell lymphomas of nasal type (NK/T-nasal type); T-LBL, T-cell lymphoblastic lymphoma; MF, mycosis fungoides; FL, follicular lymphoma; MCL, mantle cell lymphoma; MZL, marginal zone lymphoma, CLL/SLL, chronic lymphocytic leukemia/small lymphocytic lymphoma; DLBCL, diffuse large B-cell lymphoma; PMLBCL, primary mediastinal large B-cell lymphoma; BL, Burkitt lymphoma; HG-NHL, low-grade non-Hodgkin lymphoma.

## Data Availability

All data can be found in the manuscript.

## Supplementary Materials

The following supporting information can be downloaded at: https://www.mdpi.com/article/10.3390/cancers14051186/s1, Figure S1: Immunostaining for STING in dendritic cells title; Figure S2: Double-immunostaining for STING and PAX5; Figure S3: Association of ALK expression with clinical outcome in the group of ALK+ and ALK- ALCL; Table S1: List of cell lines used in the present study.
