# Supplementary material for: Sting Is Commonly and Differentially Expressed in T- and Nk-Cell but Not B-Cell Non-Hodgkin Lymphomas
Source: Cancers (Basel). 2022 Feb 24;14(5):1186. doi: 10.3390/cancers14051186 (PMC8909177; doi:10.3390/cancers14051186)
Supplement: Supplementary file 1 [file cancers-14-01186-s001.zip › cancers-1550106-supplementary.pdf]

SUPPLEMENTAL FIGURES

Figure S1. Immunostaining for STING in dendritic cells

STING was sttrongly expressed in dendritic cells in reactive lymph node and tonsilar tissue. CD21 served as a marker of follicular dendritic cells in reactive germinal centers. *Insets*: High magnification showing typical morphology of individual follicular dendritic cells positive for both CD21 and STING.

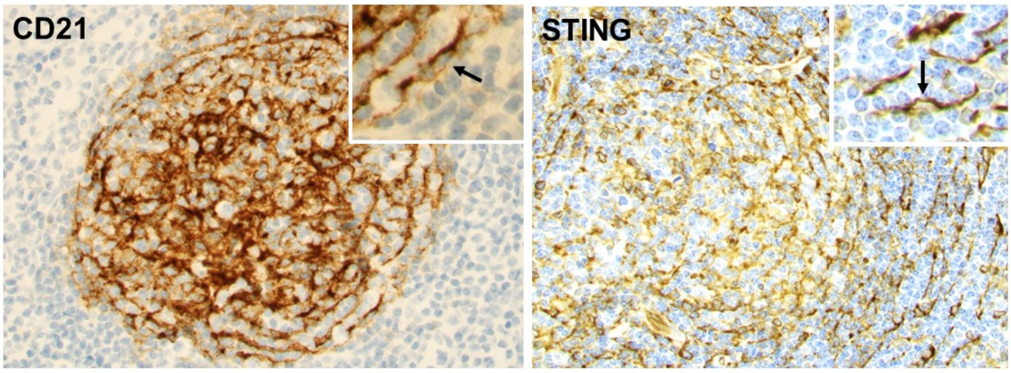

Figure S2. Double-immunostaining for STING and PAX5

Double-immunostaining for STING and PAX5 was performed in a follicular lymphoma tissue microarray, which included duplicate tumor cores from 60 tumors. STING expression is shown with red and PAX5 expression with dark brown color. Two representative cases (A, B) are shown. All follicular lymphoma cells are positive for PAX5 and negative for STING (arrows). Dendritic cells and a subset of reactive T-lymphocytes are positive for STING and negative for PAX5.

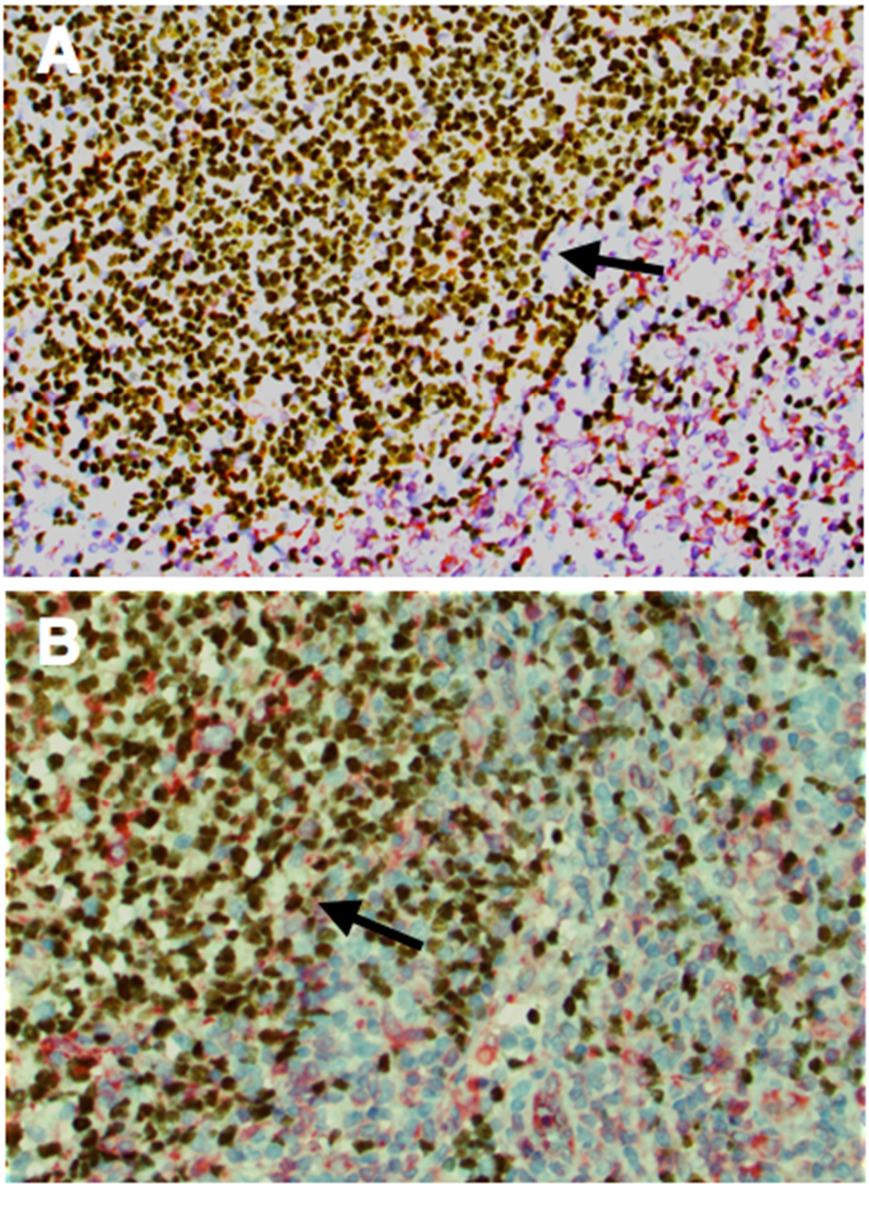

Figure S3. Association of ALK expression with clinical outcome in the group of ALK+ and ALK- ALCL

A. Freedom from progression (FFP)

B. Overall survival (OS)

A.

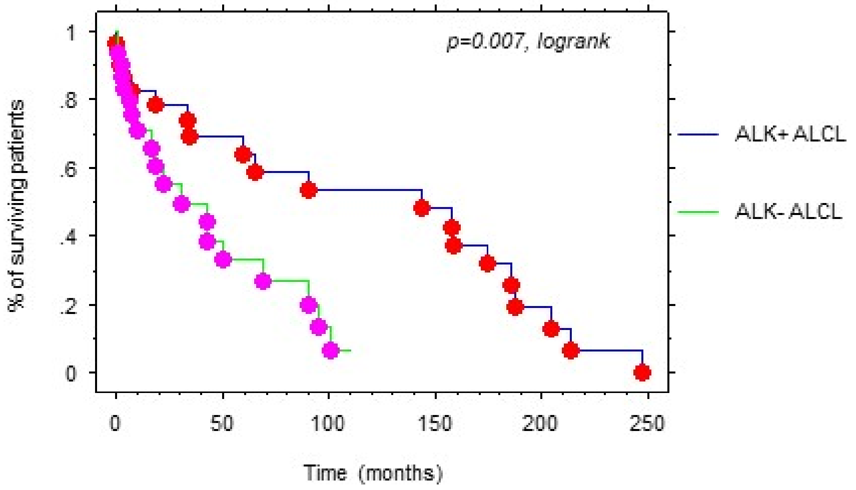

B.

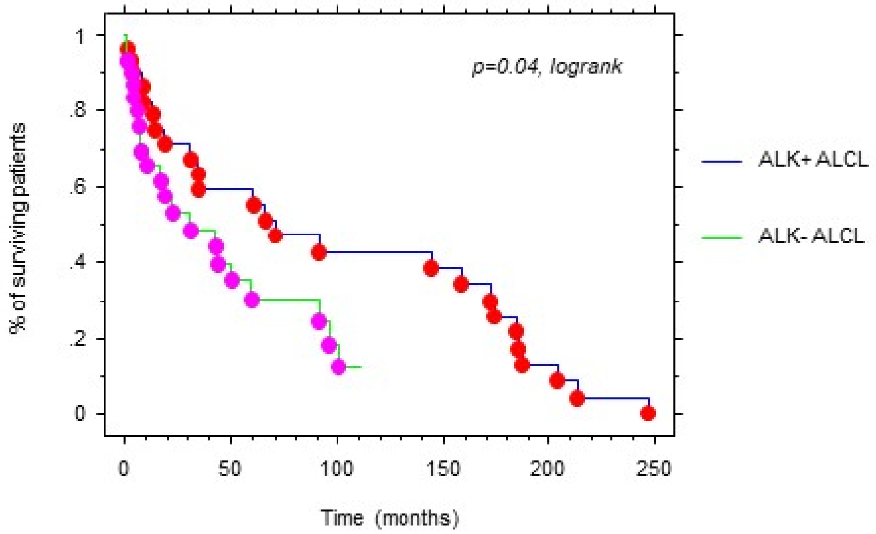

SUPPLEMENTAL TABLE

Table S1. List of cell lines used in the present study

Cell line	Lymphoma type	ALK status
**Jeko-1 ***	Mantle cell lymphoma	Negative
**JVM2 ***	Mantle cell lymphoma	Negative
**Granta519 ***	Mantle cell lymphoma	Negative
**Z138 ***	Mantle cell lymphoma	Negative
**MS**	Diffuse large B-cell lymphoma	Negative
**RCK8**	Diffuse large B-cell lymphoma	Negative
**SUDHL4**	Diffuse large B-cell lymphoma	Negative
**Karpas 299 ****	Anaplastic large cell lymphoma	Positive
**DEL #**	Anaplastic large cell lymphoma	Positive
**SUP-M2 #**	Anaplastic large cell lymphoma	Positive
**L82 #**	Anaplastic large cell lymphoma	Positive
**Mac-1 ****	Anaplastic large cell lymphoma	Negative
**Mac-2A ****	Anaplastic large cell lymphoma	Negative
**Hut78**	Cutaneous T-cell lymphoma	Negative

Purchased from ATCC, USA
* A gift from Prof. B. Sander, Karolinska Institute, Stockholm, Sweden
** A gift from Dr. Marshal Kadin (Boston, MA, USA)
# Purchased from DSMZ, Germany
